# Clinical response in Japanese metastatic melanoma patients treated with peptide cocktail-pulsed dendritic cells

**DOI:** 10.1186/1479-5876-3-4

**Published:** 2005-01-28

**Authors:** Yasuto Akiyama, Ryuji Tanosaki, Naoki Inoue, Makiko Shimada, Yukie Hotate, Akifumi Yamamoto, Naoya Yamazaki, Ichiro Kawashima, Ikuei Nukaya, Kazutoh Takesako, Kouji Maruyama, Yoichi Takaue, Ken Yamaguchi

**Affiliations:** 1 Immunotherapy Division, Shizuoka Cancer Center Research Institute, Shimonagakubo, Nagaizumi-cho, Shizuoka, Japan; 2 Stem Cell Transplantation Unit, National Cancer Center Hospital, 5-1-1 Tsukiji, Chuo-ku, Tokyo, Japan; 3 Biotechnology Research Laboratories, Takara Bio Inc., Ltd, Seta 3-4-1, Otsu, Shiga, Japan

## Abstract

**Background:**

Metastatic, chemotherapy-resistant melanoma is an intractable cancer with a very poor prognosis. As to immunotherapy targeting metastatic melanoma, HLA-A2^+ ^patients were mainly enrolled in the study in Western countries. However, HLA-A24^+ ^melanoma patients-oriented immunotherapy has not been fully investigated. In the present study, we investigated the effect of dendritic cell (DC)-based immunotherapy on metastatic melanoma patients with HLA-A2 or A24 genotype.

**Methods:**

Nine cases of metastatic melanoma were enrolled into a phase I study of monocyte-derived dendritic cell (DC)-based immunotherapy. HLA-genotype analysis revealed 4 cases of HLA-A*0201, 1 of A*0206 and 4 of A*2402. Enriched monocytes were obtained using OptiPrep™ from leukapheresis products, and then incubated with GM-CSF and IL-4 in a closed serum-free system. After pulsing with a cocktail of 5 melanoma-associated synthetic peptides (gp100, tyrosinase, MAGE-2, MAGE-3 and MART-1 or MAGE-1) restricted to HLA-A2 or A24 and KLH, cells were cryopreserved until used. Finally, thawed DCs were washed and injected subcutaneously (s.c.) into the inguinal region in a dose-escalation manner.

**Results:**

The mean percentage of DCs rated as lin^-^HLA-DR^+ ^in melanoma patients was 46.4 ± 15.6 %. Most of DCs expressed high level of co-stimulatory molecules and type1 phenotype (CD11c^+^HLA-DR^+^), while a moderate number of mature DCs with CD83 and CCR7 positive were contained in DC products. DC injections were well tolerated except for transient liver dysfunction (elevation of transaminases, Grade I-II). All 6 evaluable cases except for early PD showed positive immunological responses to more than 2 melanoma peptides in an ELISPOT assay. Two representative responders demonstrated strong HLA-class I protein expression in the tumor and very high scores of ELISPOT that might correlate to the regression of metastatic tumors. Clinical response through DC injections was as follows : 1CR, 1 PR, 1SD and 6 PD. All 59 DC injections in the phase I study were tolerable in terms of safety, however, the maximal tolerable dose of DCs was not determined.

**Conclusions:**

These results suggested that peptide cocktail-treated DC-based immunotherapy had the potential for utilizing as one of therapeutic tools against metastatic melanoma in Japan.

## Background

Despite many attempts in the last few years to target cancer-specific antigens, a breakthrough in terms of clinical response has yet to be achieved mainly because of a scarcity of effective genuine cancer antigens, immunological evasion, or an immunosuppressive state.

Melanoma-associated antigens are categorized as class I human leukocyte antigen (HLA)-restricted cancer/testis antigens [1] which are considered to be tolerable to the immune system because they are also expressed in normal tissues. However, malignant melanoma is the most well known cancer in which multiple tumor-specific antigens have been defined and utilized in vaccination strategies as peptide vaccines or peptide-pulsed DC vaccines [2-9].

From a clinical point of view, several vaccination strategies for stage IV melanoma using a combination of several (more than 3) peptides with a restriction to HLA-A2 have been reported to date [10,11]. However, little immunotherapeutic study regarding HLA-A24-restricted multiple peptides has been conducted because HLA-A24 is not a common allele in Caucasians. Several studies have demonstrated the identification of many HLA-A24-restricted CTL epitopes from various cancer-related antigens including p53, CEA, telomerase, tyrosinase, MAGE proteins etc. [12-18]. When it comes to melanoma, our group demonstrated the feasibility of using a combination of 5 melanoma-associated peptides with restriction of HLA-A24 (peptide cocktail) as a specific cancer vaccine in an immunotherapeutical trial (Akiyama et al, Anticancer Res., 2004). Based on basic research results, a phase I clinical trial of HLA-A2 or A24-restricted melanoma peptide cocktail-pulsed dendritic cell-based immunotherapy has been performed. Here we describe the safety and efficacy of DC-based immunotherapy against metastatic melanoma.

## Materials and methods

### Patient characteristics and eligibility criteria

Nine patients with metastatic melanoma were enrolled in a phase I clinical trial of a peptide cocktail-pulsed DC-based vaccine approved by the Institutional Review Board (No. 12–93 and 12–94) of the National Cancer Center, Tokyo. All patients gave written informed consent. All patients had received prior surgery, chemotherapy and radiation (Table 1). Three subjects had metastatic lesions in the brain and been given radiation to control them. Inclusion criteria were: i) biopsy-proven stage IV metastatic melanoma, ii) age ≥ 18 years, iii) performance status ≤ 2, iv) HLA-A2 or A24 phenotype and v) measurable target lesions. Exclusion criteria were : i) prior therapy < 4 weeks before trial entry, ii) untreated CNS lesion, iii) pregnancy, iv) autoimmune disease, and v) concurrent corticosteroid/immunosuppressive therapy. All the patients, who gave written informed consent, received subcutaneously (s.c) 3 DC vaccines at the inguinal region weekly and toxicity was checked. DCs were injected in dose-escalation design at a dose level per cohort of 1.0, 2,0 and 5.0 × 10^7^/body/shot (Table 1). The injected DC number was calculated from the percentage of Lin^-^HLA-DR^+ ^gated populations in a FACS analysis.

**Table 1:** Phase I study of DC-based therapy against melanoma

**Patient No.**	**Age**	**Sex**	**Previous therapy**	**Measurable lesions**	**DC injection (times)**	**Side effect**	**DTH**		**Response**
							**peptide**	**KLH**	
1	41	F	ST, CT, RT, IFNβ	lung, LN	1 × 10^7^(10)	Hepatic (II)	-	++	PR
2	75	M	ST, CT, IFNβ	LN	1 × 10^7^(10)	-	+	+	SD
3	49	F	ST, CT, IFNβ, RT	lung, liver	1 × 10^7^(3)	-	-	-	(PD)*
4	49	M	ST, CT	lung, liver	2 × 10^7^(6)	-	-	-	PD
5	50	M	ST, CT, IFNβ	lung, liver, LN	2 × 10^7^(6)	Hepatic (I)	-	-	PD
6	69	M	ST, CT, IFNβ	LN	2 × 10^7^(10)	-	+	+	CR
7	61	M	ST, CT, RT	liver, LN	5 × 10^7^(8)	Hepatic (I)	+	++	PD
8	64	F	ST, CT, RT	lung	5 × 10^7^(3)	Fever (I)	-	-	(PD)
9	66	F	ST, CT,	lung, LN	5 × 10^7^(3)	-	-	-	(PD)

* The (PD) patients represent those who received fewer than 4 DC injections because of an early progression of the disease.

### Preparation of DCs and peptides

Leukapheresis products from 7 L of processed blood were washed and centrifuged using density-adjusted OptiPrep™ (Axis-Shield PoC, Oslo, Norway), then the monocyte layer at the top was retrieved. Cells were transferred to an X-fold culture bag (Nexell, Irvine, CA) and cultured in the presence of GM-CSF at 50 ng/ml (CellGenix, Freiburg, Germany) and IL-4 at 50 ng/ml (CellGenix) in X-VIVO15 serum-free medium (Biowhittaker, Walkersville, MD). After 7 days, harvested cells were pulsed with a cocktail of 5 melanoma-specific synthetic peptides (25 μg/ml each) restricted to HLA A2 or A24 and KLH (25 μg/ml, Intracell, Frederick, MD). DC-enriched cells were washed and cryopreserved in Cryocyte bags (Baxter Healthcare Co., Deerfield, IL) until used. The purity of CD14^+ ^cells was evaluated with a flow cytometer (FACSCalibur, Becton-Dickinson Co., CA) before and after OptiPrep™ separation. The percentage of DCs was rated as the lin^-^HLA-DR^+ ^population (lineage antibodies including CD3, CD14, CD16, CD19, CD20, CD56 ; Becton-Dickinson Co.). The additional DC-related markers were determined on gated lin^-^HLA-DR^+ ^cells. The following peptides restricted to HLA-A2 or A24 were synthesized according to GMP standards by Multiple Peptide Systems, CA. HLA-A2: MART-1_27–35 _(AAGIGILTV), gp100_209–217 _(IMDQVPFSV), tyrosinase_368–376 _(YMDGTMSQV), MAGE-2_157–166 _(YLQLVFGIEV), MAGE-3_271–279 _(FLWGPRALV) ; HLA-A24: gp100_152–160 _(VWKTWGQYW), tyrosinase_206–214 _(AFLPWHRLF), MAGE-1_135–143 _(NYKHCFPEI), MAGE-2_156–164 _(EYLQLVFGI), MAGE-3_195–203 _(IMPKAGLLI).

### Characterization of tumor specimens before DC vaccines

Skin metastatic lesions were obtained from patients who gave written informed consent. The expression of melanoma tumor antigens was investigated using RT-PCR as described previously [19]. HLA protein expression was also evaluated using an immunohistochemical (IHC) analysis with anti-HLA-A2 or A24 monoclonal antibody (One Lambda Inc., Canoga Park, CA). A phenotypical analysis of lymphocytes infiltrating the tumor site was also performed using IHC.

### Clinical and immunological monitoring

Adverse effects were evaluated according to the NCI Common toxicity criteria after 3 DC injections. Standard conventional definitions of major (complete or partial) objective responses were used. Stable disease (SD) was defined as less than a 25% change in size with no new lesions lasting at least 4 weeks. Clinical response was rated as maximal through the DC vaccinations. The patients received up to 10 injections on the condition that at least one measurable lesion showed more than stable disease (SD) response and/or an ELISPOT assay performed after 4 injections indicated a positive response for more than 1 melanoma-associated peptides. PBMC samples were harvested before and 29, 78, 134 and 190 days after the 1 st DC injection, and frozen prior to use for immunological monitoring tests. All patients were followed up for 2 years after the enrollment into the study.

### ELISPOT assay

The ELISPOT assay was performed using in vitro re-stimulations. Briefly, PBMCs were incubated in a 24-well culture plate at 4 × 10^6 ^per ml and divided into non-adherent and adherent cells. Adherent cells were treated with a peptide cocktail and β2-microglobulin for 2 hrs, and co-cultured with non-adherent cells in the presence of IL-2 at 15 U/ml and IL-7 at 10 ng/ml. On day 7, non-adherent cells were re-stimulated with peptide-pulsed adherent cells. On day14, responder cells (1 × 10^4^/well) were incubated with peptide-pulsed target cells (1 × 10^5^/well; .221A201 cells for HLA-A2 peptide or TISI cells for HLA-A24 peptide) in a 96-well culture plate coated with anti-IFN-γ antibody (MABTECH AB, Nacka, Sweden) overnight. Finally positive spots stained with anti-IFN-γ antibody were measured using the KS ELISPOT system (Carl Zeiss AG, Overkochen, Germany). HLA-A2-restricted Influenza M1 peptide (GILGFVFTL) or HLA-A24-restricted EBNA3A peptide (RYSIFFDY) was used as a negative control.

### Tetramer staining

PBMCs were re-stimulated twice in vitro and utilized for tetramers staining. CD8^+^-enriched T cells were obtained by the depletion of CD4^+ ^T cells using Dynabeads M-450 CD4 (Dynal, Oslo, Norway) and used for tetramers staining. The staining was performed according to the method reported by Kuzushima et al [20]. The PE-labeled tetramers used in the present study were as follows: HLA-A*0201 MART1 (Beckman Coulter Inc., San Diego, CA), HLA-A*0201 gp100, HLA-A*2402 tyrosinase, HLA-A*2402 MAGE-1, HLA-A*2402 HIV (RYLRDQQLL) and HLA-A*0201 Influenza M1 tetramers (MBL, Nagoya, Japan).

### Intracellular cytokine staining

PBMCs were stimulated with 25 ng/ml of PMA (Sigma) and 1 μg/ml of ionomycin (Sigma) for 5 hrs in a 96-well culture plate. Breferdin A (10 μg/ml) was also added to cultures in the last hour. After the stimulation, cells were stained with FITC-anti-CD4 MoAb, and subsequently intracellular staining was performed with fix/permealization buffer and PE-labeled anti-IFN-γ or anti-IL-4 MoAb (Pharmingen, San Diego, CA). Finally, the ratio of Th1 (IFN-γ^+^) and Th2 (IL-4^+^) was calculated in PBMC samples obtained before and after DC vaccination.

### DTH reactions

The HLA-A2 or A24 peptide cocktail solution diluted to a dose of 5 μg/ml (each peptide) and KLH (50 μg/ml) were injected intradermally on the patient's forearm and the redness and induration at the injection site was measured. PPD was used as a positive control.

### Statistical analysis

Statistical differences were analyzed using Student's paired two-tailed *t*-test. Values of *p *< 0.05 were considered significant.

## Results

### DC characterization

The mean percentage of DCs rated as lin^-^HLA-DR^+ ^in melanoma patients was 46.4 ± 15.6 %, not different from that in healthy volunteers (data not shown). The frequencies of the DC-related markers were determined on gated lin^-^HLA-DR^+^cells : HLA-class I 97.5 ± 0.9 %, CD80 87.6 ± 6.9 %, CD86 85.5 ± 7.4 %, CD1a 55.2 ± 24.2 %, CD83 29.9 ± 13.3 %, CCR7 32.4 ± 13.7 %, DC SIGN 78.2 ± 19.3 %, CD11c^+^HLA-DR^+ ^90.6 ± 6.0 %, CD123^+^HLR-DR^+ ^0.99 ± 1.3 %. Most of DCs expressed high level of co-stimulatory molecules and type1 phenotype (CD11c^+^HLA-DR^+^), while a moderate number of mature DCs with CD83 and CCR7 positive were contained in DC products. On the other hand, the T cell-stimulating activity of DCs investigated in the MLR assay using allogeneic T cells was as strong as that of DCs obtained from healthy volunteers (data not shown).

### Characterization of tumor specimen

**Figure 1:**
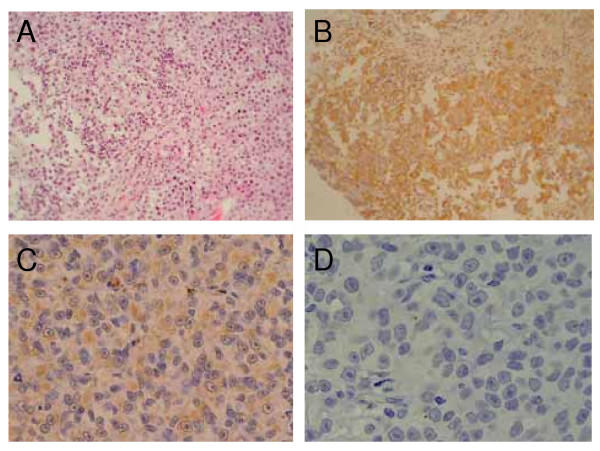
Immunohistochemical analysis of metastatic tumor tissue from responder patient 1 and non-responder patient 7. A; H-E stain and B; anti-HLA-A24 MoAb from patient 1. C; anti-HLA-A2 MoAb before DC vaccination and D; anti-HLA-A2 MoAb after 4 DC injections from patient 7. Magnification × 200.

An analysis of melanoma antigen expression by RT-PCR was performed in 3 cases. The expression of more than 2 antigens in the tumor was verified in all cases. HLA protein expression was positive in 5 out of 9 cases (Table 2). Patient 1 who showed a remarkable clinical response (PR), was representative of HLA protein-positive cases (Fig. 1). In contrast, in patient 7, HLA-A2 protein expression in the tumor was lost in the course of treatment.

**Table 2:** Immunological monitoring in melanoma patients

**Patient No.**	**HLA**	**Tumor antigen, HLA expression**	**ELISPOT**	**Tetramer**	**Th1/Th2 balance**
1	A*2402	3/5(Tyr,M1,M2), A24(+)	3/5(Tyr,M1,M2)	Tyrosinase (0.34%)	5.19 (1.45)^a^
2	A*0201	A2(+)	2/5(MART1,gp100)	MART1 (0.64%)	3.68 (1.49)
3	A*2402	A24(-)	N. D^b^	N. D.	-
4	A*0206	A2(-)	2/5(MART1, M2)	-	3.05 (2.57)
5	A*0201	A2(-)	2/5(MART1, M2)	MART1 (1.48%)	2.83 (3.68)
6	A*2402	2/5(M2,M3), A24(+)	2/5(M2, M3)	-	3.76 (2.00)
7	A*0201	4/5(MART1,Tyr,gp100,M2), A2(+)	2/5(gp100, M2)	-	2.64 (1.79)
8	A*2402	A24(-)	N. D.	N. D.	-
9	A*0201	A2(+)	1/5(gp100)^c^	N. D.	N. D.

^a^The value in the parenthesis shows Th1/Th2 ratio prior to DC vaccination. ^b^N. D. ; not done.

^c^The value shows the one obtained prior to DC vaccines.

### ELISPOT assay

**Figure 2:**
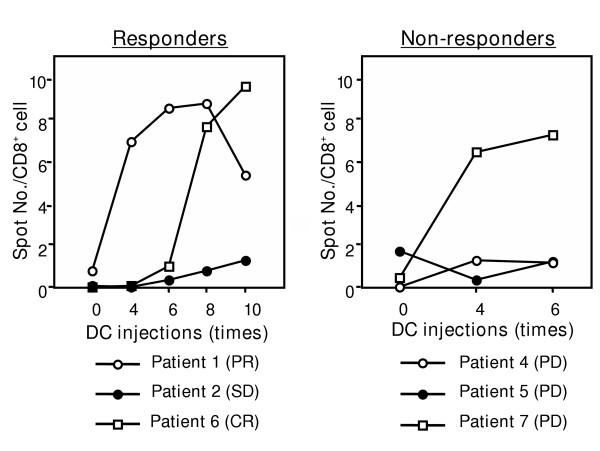
CTL responses in the course of DC injections in 6 evaluable cases. Patients 1, 2 and 6 were responders and patients 4, 5 and 7 were non-responders. Responders (cases 1,6) showed remarkable CTL expansion in PBLs compared with before DC vaccination. In contrast, non-responders (patients 4,5) showed no significant CTL responses except in patient 7.

**Figure 3:**
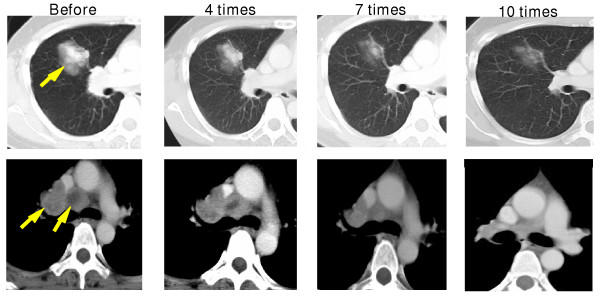
Impact of DC vaccines on metastatic lesions of the lung in responder patient 1. Upper and lower panels show a lung and hilar lymph node metastatic lesion (arrow), respectively. The CT scan was made before therapy and after 4, 7 and 10 DC vaccinations.

CTL precursors of more than 2 melanoma peptides were recognized after DC vaccines in 6 of 9 cases. Two HLA-A2^+ ^cases (patients 5 and 9) showed HLA-A2 peptide-specific CTL responses before the vaccination. Patients 1 and 6, which showed remarkable clinical responses, exhibited many CTL precursors against a HLA-24 restricted peptide-cocktail (Table 3, Figure 2). Notably, in patient 1, a remarkable increase in the CTL response to the HLA-A24 peptide cocktail was seen in accordance with the regression of metastatic tumor of the lung (Fig. 3). On the other hand, patient 7 also demonstrated a high CTL precursor frequency, but showed no significant clinical response.

**Table 3:** Peptide cocktail-specific CTL precursor frequency during DC vaccination

		**Spot No./CD8**^+^** T cell (%)^a^**				
**Patient No.**	**DC injection (times)**	**before**	**day29**	**day78**	**day134**	**day190**
1	1 × 10^7^(10)	1.19/0.45	6.96/0.06	8.82/0.63	8.81/0.08	5.4/0.08
2	1 × 10^7^(10)	0.07/0.05	0.07/0.2	0.02/0	0.02/0	0.29/0.03
3	1 × 10^7^(3)	N.D.^b^	N.A.^c^	N.A.	N.A.	N.A.
4	2 × 10^7^(6)	0.39/0.53	1.29/0.03	1.12/0	N.A.	N.A.
5	2 × 10^7^(6)	1.74/0.05	0.51/0.2	1.25/0.04	N.A	N.A.
6	2 × 10^7^(10)	0.21/0.27	0.31/0.28	1.18/0.24	7.80/0.19	9.82/0.30
7	5 × 10^7^(8)	0.62/0.20	6.52/0.1	7.33/0.11	N.A.	N.A.
8	5 × 10^7^(3)	N.D.	N.A.	N.A.	N.A.	N.A.
9	5 × 10^7^(3)	3.09/1.24	N.D.	N.A.	N.A.	N.A.

The percentages represent IFN-γ-positive spot No. divided by total CD8^+ ^cell No. from 1 × 10^4 ^PBMCs. ^a^Each value represents the percentage with peptide cocktail/without peptide cocktail. ^b^N.D. ; not done, ^c^N.A. ; sample not available.

### Tetramer staining

After CD4^+ ^T cell depletion, the frequency of CD8^+ ^cells was more than 85%. The proportion of PE-labeled tyrosinase-HLA-A24 tetramer-positive cells among gated CD8^+ ^cells was 0.34% in patient 1 (Table 2). HIV-A24 tetramer (negative control)-positive cells were not detected. The percentage of PE-labeled MART1-HLA-A2 tetramer-positive cells was 0.64% and 1.48% in patients 2 and 5, respectively. On the other hand, that of Influenza M1-HLA-A2 tetramer (negative control)-positive cells was 0.04%.

### Th1 and Th2 balance after DC vaccination

In 5 of 6 evaluable cases, the balance of Th1 and Th2 shifted more to Th1 after 4 DC injections compared with prior to vaccination. (Table 2). The amplitude of the shift seemed to be larger in clinical responders (patients 1, 2, 6) than non-responders (patients 4, 5, 7) (% of ratio increase; 264 ± 86 vs. 114 ± 35).

### DTH

Three of 6 evaluable cases showed positive DTH to a peptide-cocktail after DC injections (Table 1). On the other hand, 4 of 6 cases developed a DTH response to KLH protein. There were stronger reactions to KLH in patients 1 and 7.

### Adverse effects of DC vaccine

Safety was assessed after 3 DC injections in all 9 cases. Three of 9 patients developed mild hepatic dysfunction (grade I-II), however it was only transient and disappeared in spite of the continuance of DC injections. Rheumatoid factor and anti-nuclear antibody were negative before the injection, but increased to 1:160 and 1:40, respectively after the injections finished in patient 1. No clinical symptoms of autoimmune disease were found in patient 1 (Table 1).

### Clinical response

Clinical response was rated as maximal through the DC vaccinations. In 6 evaluable cases except for 3 cases of early PD cases due to a rapid progression of the disease, 1CR (patient 6), 1PR (patient 1), 1SD and 3 PD were obtained (Table 1). Large metastatic lesions in the lung and hilar nodes in patient 1 dramatically decreased in size after 4 DC injections, and almost disappeared after treatment finished (Fig. 3). Moderate sized cervical metastatic lesions in patient 6 finally started to decrease after 8 DC injections and disappeared surprisingly rapidly after the finish of DC therapy. In contrast, patient 7 who exhibited good immunological responses in the ELISPOT assay and DTH, showed no shrinkage of the tumor, resulting in cessation after 6 DC injections.

### Characterization of infiltrated lymphocytes in the tumor

**Figure 4:**
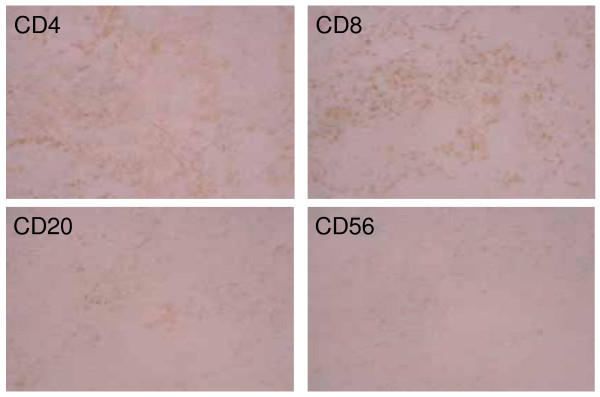
Phenotype analysis of lymphocytes infiltrating the tumor site in responder patient 1. Obvious infiltration of a larger number of CD4^+ ^or CD8^+ ^T cells and a small number of CD20^+ ^B cells is shown. Indirect staining using anti-CD4, CD8, CD20 or CD56 MoAb as primary Ab and goat anti-mouse Ab as secondary Ab was performed. Magnification × 200.

IHC analysis of infiltrated lymphocytes in the tumor after DC vaccines was performed only in patient 1 and 7. The obvious infiltration of a larger number of CD4^+ ^or CD8^+ ^T cells and a small number of CD20^+ ^B cells were shown in patient 1 (Fig. 4). In contrast, no significant cell infiltration was seen in patient 7 who did not develop any therapeutical effect on the tumor (data not shown).

## Discussion

Clinical trials of specific immunotherapy against metastatic melanoma using peptide-pulsed Mo-derived DCs have been performed in mainly Western countries, and some fruitful results were obtained [7,10,11]. In those cases, most of the patients belonged to the HLA-A*0201 type. In the present study, we investigated the effect of peptide-pulsed DCs on 4 cases of HLA-A*2402^+ ^metastatic melanoma patients besides 4 cases of HLA-A*0201^+ ^patients in a clinical phase I trial. This is the first report to demonstrate that peptide-pulsed DCs were effective in some HLA-A24^+ ^melanoma patents in Japan. It is well known that HLA-A*2402 is a common genotype and around 60% positive in Asians. There was one case of HLA-A*0206 patient among 5 HLA-A2^+ ^patients (Table 2). Sidney et al. [21] demonstrated that over 70% of the peptides that bound A0201 with high affinity were found to bind at least two other supertype molecules like A*0202, A*0203 or A*0206. Taking it into considerations, the HLA-A*0206 patient was finally enrolled into the study. With regard to other HLA-A24^+ ^solid cancers, stomach, colon and bladder cancers have been treated with peptide (MAGE-3)-pulsed DC vaccines, and showed a limited response [22-24]. Considering that melanoma is highly immunogenic and probably a good model for tumor-specific immunotherapy despite being an unusual tumor in Asian countries, it deserves a phase I study using peptide-pulsed DCs.

In our study, peptide cocktails combining 5 peptides for each HLA type (HLA-A2 or A24) were prepared and used for DC pulsing. Our clinical study revealed positive ELISPOT responses against more than 2 peptides in all 6 evaluable cases. In previous reports, clinical DC therapy using more than 3 melanoma peptides demonstrated the induction of a specific CTL response against multiple melanoma peptides [10,11]. However,, there is still some controversy over the efficacy of multiple epitope-based vaccinations and Smith *et al. *[25] demonstrated that, although polyepitope vaccines are an effective way of priming polyvalent CTLs, continual stimulation with polyepitope vaccines might restrict CTL induction as a result of immunodominance. The results of our study are thought to answer that question, but testing of the peptide cocktail vaccine in more patients will be needed.

To refine the quality and protocol of the tumor-specific immunotherapy for clinical trials, the prediction of clinical response in an individual is important [26] and should be discussed. In our study, the correlation between immunological parameters and clinical response was investigated in a limited number of cases.

First of all, as to HLA expression in the tumor, patients 1, 2, 6 and 7 were positive, and patients 4 and 5 were negative. HLA-negative cases showed a progression of the tumor. Even in positive cases, patient 7 turned negative in the course of DC therapy, showing tumor progression. Loss of HLA expression in melanoma is reported to be a complex phenomenon associated with melanoma antigen loss [27], β2-microglobulin gene mutation [28] or loss of heterozygosity (LOH) in chromosome 6 and may lead to tumor progression and metastasis. As to patient 7, considering that the melanoma antigen expression was maintained, the functional expression of β2-microglobulin should be investigated. All the other HLA-positive cases showed CR, PR and SD, respectively. There was a tendency for HLA expression to be associated with tumor response, and some researchers reported a positive correlation of HLA-expression to tumor response in immunotherapy against melanoma. However, despite the positive correlation of HLA-expression in the tumor with anti-tumor response, Nestle et al. demonstrated that HLA-expression in the tumor did not correlate to survival in melanoma patients [29].

Second, the amplitude of the CTL response in the ELISPOT assay seems to be another key factor predicting anti-tumor response. Patients 1, 6 and 7 showed large responses to peptide cocktail in ELISPOT, and patients 2, 4 and 5 showed small responses. The former exhibited a remarkable regression of tumor except patient 7. On the other hand, the latter showed a poor response. There was a likely tendency that the amplitude of the CTL response was associated with tumor regression. Also, it was difficult to predict when immunological responses like CTL induction start to be activated in vivo during DC vaccination, and this question needs to be answered. In the present study, because of a limited number of patients given DC vaccines, the tendency that HLA-class I protein expression in the tumor and the amplitude of ELISPOT responses are seemingly associated with tumor regression is not convincing.

Finally, in order to improve tumor response in the present study, there are still some issues regarding clinical DC preparation. First of all, the purity of CD14^+ ^cells after Opti-prep separation is still low and may not be reproducible. Therefore, other clinical grade-monocyte separation methods using an elutriator or negative selection with CD2 and CD19 MoAbs [30] should be tried. Second, considering that the amplitude of the CTL response was associated with tumor regression, and that even a remarkable increase of CTL frequency inevitably diminished in spite of the repetition of DC vaccinations, it seems to be crucial to maintain increased CTL frequency in blood leading to TIL in the tumor and expand more than enough to develop a substantial number of memory CD8^+ ^CTL in lymph nodes. Such a novel method will be needed to develop an effective cancer vaccine.

## Conclusions

In the present study, we investigated the effect of dendritic cell (DC)-based immunotherapy on metastatic melanoma patients with HLA-A2 or A24 genotype. Nine cases of metastatic melanoma were enrolled into a phase I study using HLA-A2 or A24-restricted peptide cocktail-pulsed DCs. All 6 evaluable cases showed positive immunological responses to more than 2 melanoma peptides in an ELISPOT assay. Clinical response through DC injections was as follows : 1CR, 1 PR, 1SD and 6 PD. All 59 DC injections in the phase I study were safely administered to patients. These results suggested that peptide cocktail-treated DC-based immunotherapy had the potential for utilizing as one of therapeutic tools against HLA-A2 or A24^+ ^metastatic melanoma.

## Abbreviations

DC, dendritic cell ; HLA, human leukocyte antigen ; GM-CSF. granulocyte macrophage-colony-stimulating factor ; IL, interleukin ; KLH, Keyhole limpet hemocyanin ; CTL, cytotoxic T cell ; DTH, delayed-type hypersensitivity ; CR, complete remission : PR, partial remission ; SD, stable disease ; PD, progressive disease ; RT-PCR, reverse transcription-polymerase chain reaction ; IFN, interferon ; PBMC, peripheral blood mononuclear cell.

## Competing interests

The authors declare that they have no competing interests.

## Authors' contributions

YA participated in the design of the study and drafting the manuscript and were responsible for completing the study. RT, NI, MS, YH carried out apheresis and cell processing and were responsible for DC production. AY and NY were responsible for the clinical side of the study. IK, IN, KT and KM participated in the design of the study and performed biological assays. YT and KY reviewed the manuscript. All authors read and approved the final manuscript.
